# Neddylation steers the fate of cellular receptors

**DOI:** 10.1038/s12276-024-01358-0

**Published:** 2024-12-02

**Authors:** Jun Bum Park, Min Young Lee, Jooseung Lee, Geon Ho Moon, Sung Joon Kim, Yang-Sook Chun

**Affiliations:** 1 Department of Biomedical Sciences, Seoul National University College of Medicine, Seoul, Republic of Korea; 2 Ischemic/Hypoxic Disease Institute, Seoul National University College of Medicine, Seoul, Republic of Korea; 3 Department of Medicine, Massachusetts General Hospital, Harvard Medical School, Boston, MA USA; 4 Department of Physiology, Seoul National University College of Medicine, Seoul, Republic of Korea

**Keywords:** Neddylation, Physiology, Neddylation

## Abstract

Cellular receptors regulate physiological responses by interacting with ligands, thus playing a crucial role in intercellular communication. Receptors are categorized on the basis of their location and engage in diverse biochemical mechanisms, which include posttranslational modifications (PTMs). Considering the broad impact and diversity of PTMs on cellular functions, we focus narrowly on neddylation, a modification closely resembling ubiquitination. We systematically organize its canonical and noncanonical roles in modulating proteins associated with cellular receptors with the goal of providing a more detailed perspective on the intricacies of both intracellular and cell-surface receptors.

## Understanding neddylation's role in receptor functionality

Proteins undergo posttranslational modifications (PTMs) to maintain physiological balance. Neddylation, a type of PTM, involves attaching a small ubiquitin-like molecule, NEDD8, to target proteins. In this study, J.B.P. and colleagues explore the role of neddylation role in cellular receptors. The researchers conducted a review to understand how neddylation affects different types of receptors, including membrane and intracellular receptors. They examined both canonical (cullin-dependent) and noncanonical pathways regulated by neddylation. The study systematically analyzes the impact of neddylation on receptor stability, signaling, and function.

This summary was initially drafted using artificial intelligence, then revised and fact-checked by the author.

## Introduction

Proteins undergo posttranslational modification (PTM), a process that contributes to various biological processes by modifying protein dynamics to maintain physiological homeostasis. Comprising over 400 different types, PTMs are commonly observed in a wide array of proteins modulating activity, location, structure, and interactions^1^. Among many PTMs, 24 major PTMs exist, with phosphorylation, acetylation, and ubiquitination being extensively studied^2^. Like ubiquitination, ongoing research is being conducted on ubiquitin-like protein modifications, including small ubiquitin-like modifier (SUMO) and neural precursor cell expressed, developmentally downregulated 8 (NEDD8). Among these proteins, NEDD8 stands out for its high sequence and structural similarity to ubiquitin, with approximately 59% identity in human orthologs^3,4^. Additionally, unlike ubiquitination, sumoylation is known to occur predominantly in the nucleus, where it is involved in nuclear processes^5–7^. Given our focus on PTMs that affect receptors in both the nucleus and the cytoplasm, NEDD8 stands out as an ideal target for study owing to its close resemblance to ubiquitin and its broad role in various cellular processes across both compartments. However, small structural differences such as a single amino acid variation at the C-terminus (Ala72 in NEDD8 and Arg72 in ubiquitin) contribute to distinct interactions that result in unique functionalities under normal physiological conditions^3,8^. Therefore, it is crucial to investigate neddylation not only as a modification that accompanies ubiquitination but also as a standalone PTM regulator contributing significantly alongside other major PTMs.

**Fig. 1:**
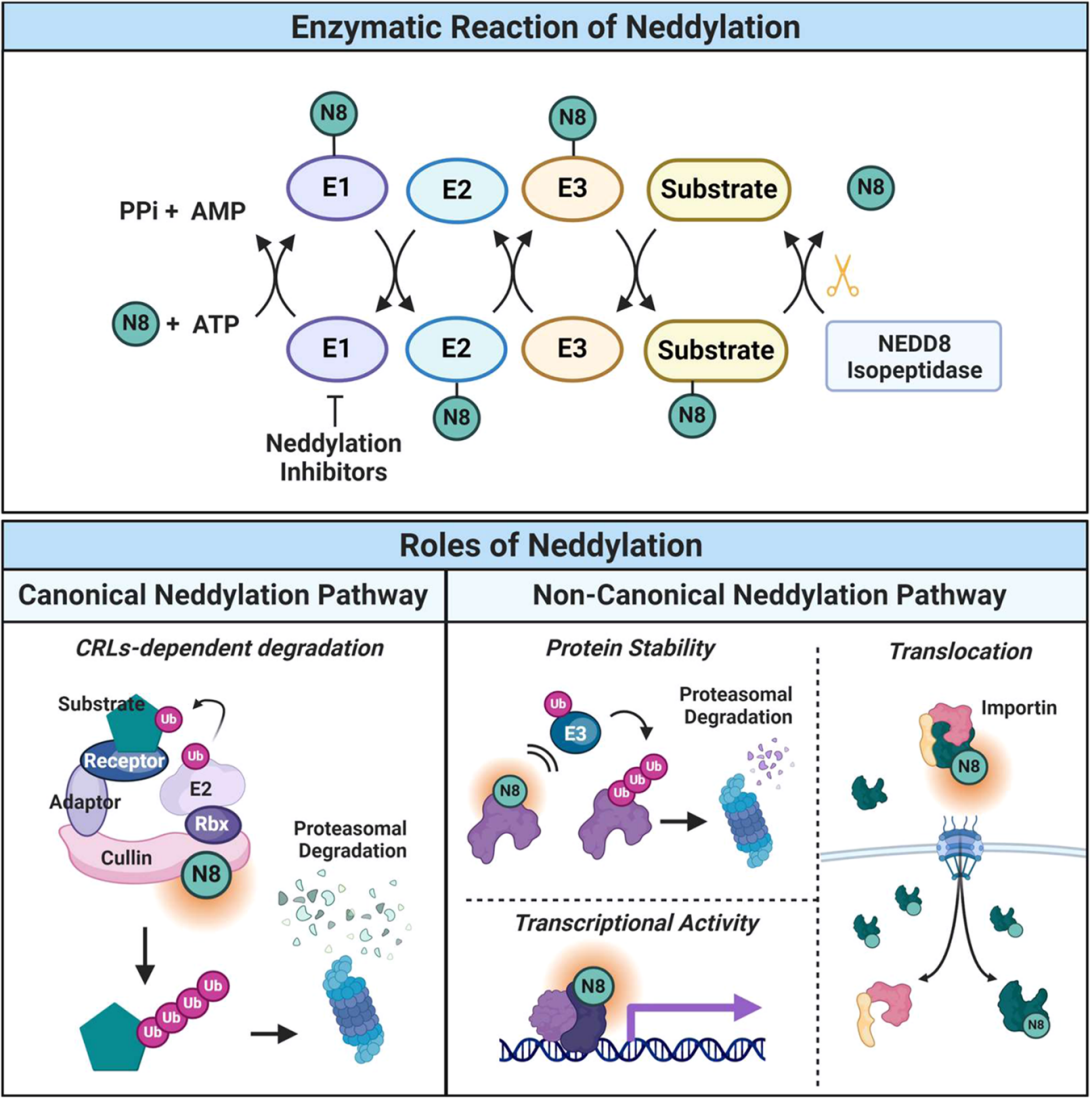
The process and roles of neddylation. Neddylation is a sequential process in which N8 is activated by the E1 enzyme, loaded onto the E2 enzyme, and conjugated to the substrate by the E3 enzyme. E1 enzyme activity can be blocked by neddylation inhibitors and E1 can be recycled through N8 isopeptidase. Neddylation can be divided into two regulated pathways, the canonical regulated pathway and the noncanonical regulated pathway, depending on how the substrates are involved. In the canonical regulated neddylation pathway, N8 conjugation to the cullin protein (a subunit of cullin-RING ubiquitin ligase) activates the CRL, leading to protein ubiquitination. The noncanonical regulated neddylation pathway occurs when N8 conjugates with substrates other than cullin proteins. Unlike the canonical neddylation pathway, noncullin-regulated neddylated proteins trigger a diverse range of effects on substrate proteins, inducing changes in their stability, function, and subcellular localization.

Neddylation is a biochemical process involving the covalent attachment of a small ubiquitin-like molecule, NEDD8, to designated target proteins. NEDD8 is activated by neddylation-activating enzyme (NAE1) and then transferred to target substrates by the E2 enzyme UBC12 and E3 ligases^9^. The regulation of neddylation can be categorized into two groups: canonical and noncanonical. Neddylation varies depending on the nature of the substrates involved. Canonical regulation by the neddylation process involves the modification of cullin family proteins with NEDD8. Cullin family members, which function as scaffold proteins for the cullin-RING ubiquitin ligase (CRL), are the most extensively studied targets of neddylation^10^. Inactive CRLs are activated through mononeddylation near the RBX binding site, prompting a conformational change that facilitates the transfer of ubiquitin molecules to their specific substrates for degradation^11^. On the other hand, noncanonical regulation by neddylation is a similar process in which NEDD8 is attached to protein substrates other than cullin molecules^12^. Noncanonical regulation by neddylation is observed in various cellular processes, such as protein synthesis, and triggers a diverse range of effects on substrate proteins, inducing modifications in their stability, functions, and subcellular localization, as shown in Fig. 1. For a more detailed explanation of the fate of neddylation, readers are encouraged to refer to a recently published comprehensive review^12^. Despite the involvement of many proteins in cellular receptors across diverse pathways, the role of neddylation in receptor proteins has received relatively little attention.

Cellular receptors play crucial roles in various processes, not only in development but also across most organ systems and in human pathophysiology. For example, proper cell development requires the precise coordination of signaling pathways such as fibroblast growth factor (FGF) and Wnt, highlighting the significant role that cellular receptors play in determining cell fate^13^. Additionally, receptors are essential for intercellular communication within organs, and their dysfunction can disrupt normal physiological processes leading to various diseases^13^. Given their importance, the study of cellular receptors has been and should continue to be a key focus in human physiology. Therefore, this review aims to present up-to-date findings on both the canonical and noncanonical regulatory roles of neddylation in major types of receptors, particularly in the mammalian system.

## Role of neddylation in cellular receptors

In most physiological processes, cells communicate with each other through the interaction of ligands and protein receptors. Regular interactions involve a single ligand binding to a single receptor, resulting in various types of cellular signaling involving different ligands and receptors. Cellular receptors can be categorized into two main categories depending on their location: membrane receptors and intracellular receptors. Membrane receptors include ion channels, G-protein receptors, and enzyme-linked protein receptors. On the other hand, there are two types of intracellular receptors: Type I or cytoplasmic receptors and Type II or nuclear receptors. Since receptors are composed of proteins, investigating the protein modifications involved in these interactions is important. With this objective in mind and to maintain conciseness, the rest of this article will focus on examining the role of neddylation for each classified receptor alongside its physiological functions, as outlined in Table 1.

**Table 1:** Neddylation-induced functional changes in receptors.

Class	Receptor	Tissue	Disease	Signaling pathway	Function of neddylation	Ref.
Membrane receptor	Ion channel					
	AchR	muscle	x	canonical	protein aggregation ▲	^23^
	NBCe1	kidney	kidney disorder		stability ▼	^19^
	NCC	kidney	kidney disorder		activity ▼	^20^
	BK channel	brain	epileptogenesis		stability ▼	^18^
	Nav1.1	brain	seizure	noncanonical	activity ▲	^22^
	Kcnj2	heart	developmental disability		mRNA level ▲	^21^
	G-protein-coupled Receptor					
	AC	brain	allodynia	canonical	activity ▲	^31^
		neuron	x		activity ▲	^32^
	ASCT2/SLC1A5	breast	cancer		stability ▼	^33^
	mGlu7	neuron	x	noncanonical	stability ▼	^36^
	VACM-1	x	x		activity ▲	^35^
	GPR85	breast	cancer	undefined	activity ▲	^38^
	CCR2	liver	fibrosis		mRNA level ▲	^37^
	Enzyme-linked Receptor					
	IRS1	neuron	learning and memory disorder	canonical	stability ▼	^42^
	IRS1, 2	liver	type 2 diabetes		stability ▲	^41^
		ovary, kidney	cancer	noncanonical	stability ▼	^45^
	EGFR	uterus	cancer		stability ▼	^47^
		skin	x		stability ▲	^48^
		lung, breast, colon	cancer		activity ▲	^49,52^
	HER2	breast	cancer		stability ▲	^50^
	TβR II	hematopoetic stem cell	leukemia		stability ▲	^46^
	GHR	x	x		stability ▼	^44^
	SIRPa	colon	cancer		activity ▼	^51^
	TLR	immune dendritic cell	x	undefined	activity ▲	^53^
Intracellular receptor	Type I (Cytoplasmic receptor)					
	ER	liver, breast	cancer	canonical	transcriptional activity ▼	^56^
		breast	cancer		stability ▼	^57^
	ERRβ	breast	cancer		stability ▼	^58^
	ER	breast	cancer	noncanonical	localization	^59^
	AR	non-human : zebrafish	x		transcriptional activity ▼	^60^
		muscle	Spinal bulbar muscular atrophy, Neurodegenerative disease		stability ▼	^61^
		prostate	cancer	undefined	transcriptional activity ▼	^54^
	Type II (Nuclear Receptor)					
	PPARγ	lipid	obesity	noncanonical	stability ▲	^63^

## Neddylation in membrane receptors

Cell-surface receptors are essential for communication between the cell and the extracellular environment. They are located within the plasma membrane of cells and receive a variety of extracellular molecules such as hormones, cytokines, growth factors, neurotransmitters, cell adhesion molecules, or nutrients^14^. Signal transduction occurs through the binding of ligands to membrane receptors, initiating intracellular responses. The general principle of signal transduction comprises four main components: protein phosphorylation, modular transduction, autoinhibitory modules, and location^15^. Drawing from this foundational principle and recognizing the importance of these main components in signal transduction, our inquiry focused on understanding how neddylation, whether directly or indirectly, influences membrane receptor proteins.

### Ion channels

**Fig. 2:**
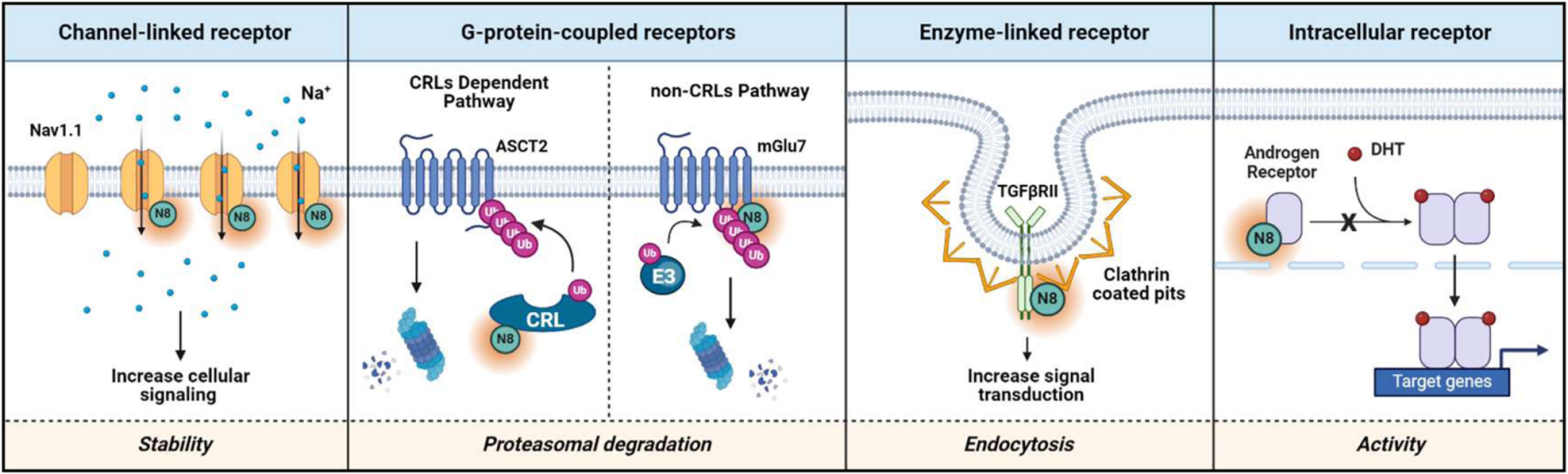
Neddylation plays diverse roles in various types of cellular receptors. Receptors are categorized into membrane and intracellular receptors based on their location. Membrane receptors include ion channel-linked, G-protein-coupled, and enzyme-linked receptors, which transmit signals from outside the cell, triggering diverse cellular responses. Intracellular receptors are divided into Type I (cytoplasmic) and Type II (nuclear) receptors, which are primarily activated by hydrophobic molecules such as hormones. They act as transcription factors, regulating various cellular responses. Neddylation has diverse effects on receptor functionality, ultimately altering cellular physiology in response to external stimuli. In some cases, neddylation enhances cellular signaling by increasing receptor stability, while in others it triggers ubiquitin–proteasomal degradation leading to reduced receptor levels and attenuated signaling. Neddylation can also enhance signal transduction by promoting receptor endocytosis. Furthermore, it diminishes the transcriptional activity of intracellular receptors by disrupting receptor‒ligand binding interactions.

Ion channels abundant in mammalian cells play crucial roles in many homeostatic functions including electrolyte transport, cellular excitability, and cell growth (as illustrated in Fig. 2). Dysfunction in the regulation and function of ion channels contributes to various diseases, characterized by alterations in ion channel function or interacting proteins^16^. There are two main types of ion channels: ligand-gated channels and voltage-gated channels. Examples of voltage-gated channels include Na^+^, K^+^, Ca^2+^, and Cl^−^ channels, while ligand-gated channels include transient receptor potential (TRP) channels, ryanodine receptors, and inositol triphosphate (IP_3_) receptors. In certain cases, the boundaries of certain K^+^ and Cl^−^ channels can be blurred, which is discussed in depth in this text^17^. As we proceeded, we compiled and organized the existing research articles for each classified receptor, highlighting the role of neddylation. These articles are categorized into canonical and noncanonical pathways of neddylation regardless of whether they are related to voltage-gated or ligand-gated channels. Additionally, we included some articles that were challenging to classify owing to their brief focus on neddylation in the noncanonical section, despite their involvement.

#### Canonical regulation by neddylation

Neddylation-associated regulation of cullins has been shown to involve ion channels, which are involved in both neurological disorders and kidney disease. Studies have reported that the degradation of the BK channel, a voltage-activated K^+^ channel, is mediated by cullin-RING ubiquitin E3 ligase 4A (CRL4A) and cereblon (CRBN) complex E3 ligase-dependent ubiquitination. This ultimately enhances channel activity and may result in epilepsy or seizures^18^. In addition to neurological disorders, ion channels are crucial for maintaining acid‒base balance in the kidney. Sodium-coupled bicarbonate cotransporter 1 (NBCe1) is pivotal in this regard, with its ubiquitin E3 ligase, NEDD4-2, reported to undergo neddylation, consequently activating and degrading the NBCe1 protein through ubiquitination^19^.

A recent study investigated an important transporter responsible for maintaining sodium and potassium balance in the body, known as the thiazide-sensitive sodium chloride cotransporter (NCC). This study revealed that cullin neddylation plays a significant role in its regulation. The authors also revealed an inverse correlation between dietary K^+^ intake and NCC activity. According to their findings, elevated levels of potassium in the bloodstream activate the process of neddylation of various cullins, including Cul1, Cul3, Cul4, and Cul5. This leads to the degradation of with-no-lysine kinases 1 and 4 (WNK1 and WNK4), reducing NCC activation and potassium reabsorption, crucial for homeostasis^20^. These references stand as the sole instances linking cullin neddylation with ion channels, indicating the need for further investigation into this process.

#### Noncanonical regulation by neddylation

Reports have also highlighted cases of noncanonical regulation by neddylation and ambiguous classifications related to neddylation in ion channels. In one study, knockout hearts lacking NAE1, a neddylation E1 enzyme, presented decreased expression of the Kcnj2 gene, which encodes a lipid-gated potassium channel. In cardiomyocytes, HIF-1α neddylation stimulates fatty acid utilization. Inhibition of NAE1 resulted in reduced Kcnj2 gene expression, highlighting the necessity of neddylation in cardiomyocyte maturation^21^. Two studies have been conducted on neurological diseases. One examined the role of neddylation in the stability of Nav1.1, an alpha subunit of the voltage-gated sodium channel. By confirming lysine mutations in epilepsy patients the authors reported that the K1936 mutation disrupted the neddylation of Nav1.1, leading to instability^22^. Furthermore, GO analysis of NAE1 knockout mice revealed that the downregulated proteins were significantly associated with neural development, neurotransmission, and synaptic plasticity^22^. Another study focused on protein aggregation, in which the E3 ligase Rapsyn is involved in the neddylation of the acetylcholine receptor (AChR), leading to cluster formation. This facilitates neuromuscular junction formation and signal transmission^23^, providing supportive evidence of the potential involvement of neddylation in various neurological cellular effects.

With clear distinctions between the canonical and noncanonical roles of neddylation, some studies investigating neddylation in ion channels have provided significant insights but lack the depth required for effective categorization within these distinctions. For example, a study of kidney distal convoluted tubules (DCTs) revealed high expression of NEDD4- and NEDD8-related proteins involved in NaCl reabsorption and potassium secretion, suggesting that neddylation-related vasopressin signaling cascades in DCTs may play important roles in regulating blood pressure^24^. Additionally, although the specific mechanism was not investigated, a report noted that neddylation blockade resulted in impaired neuronal morphology and that neurotransmitter actions through AMPA and NMDA receptors were not visible^25^. Considering all reports on the regulation of ion channels in terms of neddylation, further investigations should be proposed to gain deeper insights into the underlying mechanisms.

### G-protein-coupled receptors

G-protein-coupled receptors (GPCRs) constitute the largest family of membrane receptors and play a vital role in mediating our physiological responses to hormones, contributing to a wide range of biological effects (Fig. 2). GPCRs are divided into six main families: the rhodopsin (Class A), secretin (Class B), glutamate (Class C), fungal mating pheromone (Class D), cAMP receptor (Class E), and frizzled/smoothened (Class F) families. Each of these families features a core structure comprising seven transmembrane (TM) α helices connected by six interhelical loops of varying lengths^26^. In the context of various receptors across different classes, dysregulation of GPCR signaling has the potential to be linked to more than 30 human diseases and syndromes^27^. Currently, drugs that target GPCRs make up approximately 34% of FDA-approved therapeutics^28^ and there are ongoing investigations into the posttranslational modifications of GPCRs^29^. However, despite this active investigation, there have been few reports regarding the involvement of neddylation.

#### Canonical regulation by neddylation

While there have been numerous reports on receptors within the GPCR family, only a few have addressed the involvement of the canonical regulation pathway. One study focused on the compartments within the E3 ligase complex in adenyl cyclase (AC)-cyclic adenosine monophosphate (cAMP) signaling, particularly concerning heterologous sensitization of the AC. Researchers have reported that treatment with MLN4924 or knockdown of CUL3, NEDD8, or RBX1 results in the disappearance of heterologous sensitization of AC, highlighting the significant roles of cullin3-RING ligases and the protein degradation pathway in this process^30^. The same author published a follow-up study demonstrating that MLN4924 treatment induced inhibitory effects on AC sensitization as observed in ethanol-induced locomotor sensitization and inflammatory pain allodynia^31^. Although the exact mechanisms remain unclear, these studies suggest that the neddylation pathway may play a critical role in AC/cAMP signaling and may be a potential target for therapeutic interventions in conditions associated with AC sensitization. A recent study conducted a thorough investigation of ubiquitylation levels caused by adenylyl cyclase by employing forskolin, a cAMP activator^32^. This study confirmed a significant increase in PKC activity, resulting in global ubiquitylation and protein neddylation. By mapping a proteome dataset of diGly-modified peptides using the Database of Ubiquitinating and Deubiquitinating Enzymes (DUDE), the authors quantified nine proteins significantly regulated across various classes of E2 and E3/CUL ligase enzymes. While the administration of MLN4924 to T cells resulted in the restoration of ubiquitylation levels, the study did not delve into further details regarding the mechanism involved. The authors concluded that neddylation is necessary for proteasome activation during high-intensity exercise using cAMP, which promotes damaged protein degradation^32^.

MLN4924 treatment can prevent the ubiquitination of ASCT2, a glutamine transporter, by deactivating the CRL3-SPOP E3 ligase. Normally, when there is a high amount of glutamine in the body the CRL3-SPOP E3 ligase prevents the accumulation of ASCT through proteasomal degradation. However, in tumor environments characterized by either glutamine abundance or glutamine deprivation, the authors observed an increase in ASCT2 levels. Specifically, glutamine deprivation triggered SPOP self-ubiquitylation and subsequent degradation. This led to an inverse correlation between SPOP and ASCT2, resulting in decreased glutamine uptake and subsequently inhibiting breast cancer survival and growth^33^. Although some studies lack detailed explanations of canonical regulation by the neddylation mechanism, the strong involvement of neddylation in various physiological responses highlights the critical importance of this area of research and the pressing need for further explanation.

#### Noncanonical regulation by neddylation

Noncanonical regulation by neddylation, along with its ambiguous associated classifications, has received less attention than canonical regulation by neddylation. Nonetheless, a study using ubiquitylome analysis revealed that FANCA and FANCC, members of the FANC protein family, are involved in the neddylation of CXCR5, a chemokine receptor. Additionally, FANCA serves as the E3 neddylation ligase for CXCR5, targeting a specific site. Despite limited exploration, this study confirmed that CXCR5 neddylation enhances wound healing in osteosarcoma through a point mutation^34^. Another report has suggested that the phosphorylation of VACM-1, the vasopressin-activated Ca^+2^ mobilizing receptor, is crucial for cellular growth. Neddylation was found to be a prerequisite for VACM-1 phosphorylation by protein kinase C (PKC), followed by Ser730 phosphorylation by protein kinase A (PKA)^35^. Another study focused on the brain revealed that MLN4924 treatment increased the stability of mGlu7 in primary neurons. The authors discovered that conjugation between mGlu7 and NEDD8 and neddylation modulated the ubiquitination of mGlu7. Additionally, they demonstrated that neddylation was necessary for the localization of mGlu7 in the presynaptic active zone, underscoring the importance of neddylation in presynaptic terminal maturation^36^.

Although the classification was uncertain, but confirmed the involvement of neddylation, a study revealed that MLN4924 reduced liver injury, apoptosis, inflammation, and fibrosis, thereby preventing hepatocyte apoptosis. Specifically, increased neddylation leads to increased caspase 3 activity during bile acid-induced apoptosis, whereas neddylation inhibition alleviates apoptosis by decreasing the mRNA levels of C-C chemokine receptor type 1 (*Ccr1)*, type 2 (*Ccr2)*, and type 5 (*Ccr5)* receptors^37^. Breast cancer cells have also been found to secrete chemokine (C-X-C motif) ligand 14 (CXCL14), a chemokine ligand for the GPR85 receptor, which activates fibroblasts through the ERK1, AKT, and neddylation pathways. This activation is supported by the levels of NEDD8 and related E1 and E2 enzymes^38^. While the reports discussed have revealed the involvement of the cullin family and NEDD8 in the GPCR family network^30,32,34^, there is a pressing need for thorough research to better understand the mechanisms associated with these receptors. This is particularly crucial in light of the significant body of research on ubiquitination within the GPCR family^39^.

### Enzyme-linked receptors

Enzyme-linked receptors represent a significant category of cell-surface receptors. These receptors are composed of transmembrane proteins with their ligand binding domain located on the outer surface of the plasma membrane. Unlike GPCRs, which activate specific G proteins, enzyme-linked receptors bind to extracellular ligands and trigger enzymatic activity on the intracellular side of the membrane (as illustrated in Fig. 2). These receptors can be classified into six main classes based on their kinase-like activity: receptor tyrosine kinases (RTKs), tyrosine kinase-associated receptors, receptor serine/threonine kinases, receptor guanylyl cyclases, histidine kinase-associated receptors, and receptor-like tyrosine phosphatases^40^. Among the cellular receptors discussed in this review, this class has received the most attention concerning neddylation. Since enzyme-linked receptors cover a wide range of drug targets, updating the latest information and identifying areas or receptors that require further investigation are crucial interim steps.

#### Canonical regulation by neddylation

Compared with reports on noncanonical regulation by neddylation, reports on the canonical regulation of enzyme-linked receptors by neddylation are lacking. Two studies investigated the neddylation of insulin receptor substrates (IRSs) in different tissues. A recent study suggested that MLN4924 treatment inhibits the degradation of IRS1 and IRS2, lowering blood glucose levels and improving insulin signaling, which is relevant for conditions such as type 2 diabetes mellitus and insulin resistance^41^.

Another study exploring the correlation between metabolic syndrome and Alzheimer’s disease revealed that the process of neddylation and subsequent activation of cullin-RING ligase complexes can lead to synaptic insulin resistance by promoting the degradation of IRS1. Consequently, inhibiting neddylation preserved synaptic insulin signaling and ameliorated memory deficits in mice with a high amyloid load^42^. In addition to the insulin receptor, distinct studies have investigated the T-cell receptor (TCR) and the growth hormone receptor (GHR). Researchers have reported that neddylation negatively regulates the TCR, leading to an increase in the interleukin-2 (IL-2) population, T-cell proliferation, and Treg development in vitro. The negative impact of neddylation on the TCR was further validated using shRNAs targeting CUL1, CUL2, and CUL3^43^. According to a study conducted on GHR, the activation of the Skp1-cullin-F-box (SCF)^TrCP^ complex depends critically on neddylation for its activity. This finding holds immense importance since the transducing repeat-containing protein (TrCP) complex, which functions as a ubiquitination E3 ligase for various substrates, is responsible for the endocytosis and ubiquitination of GHR via its WD40 domain^44^. Given the many reports on noncanonical regulated enzyme-linked receptors and the variety of enzyme-linked receptor types, extensive research on cullin-dependent neddylation is essential for gaining invaluable insights.

#### Noncanonical regulation by neddylation

The noncanonical regulation of enzyme-linked receptors by neddylation has been studied extensively and has emerged as a promising avenue for research. Through the E3 ligase C-CBL, several studies have investigated the role of neddylation in different receptors. We recently discovered a crucial and novel role for C-CBL as a neddylation E3 ligase that targets both IRS1 and IRS2. Conjugation of NEDD8 to IRS1 and IRS2 induced degradation of the proteins, a process confirmed through NEDD8 knockdown and MLN4924 treatment. The increased stability of IRS proteins leads to increased AKT signaling, ultimately inducing cancer cell migration^45^. In another study, the TGF-B type 2 receptor (TBR2) was reported to undergo neddylation by C-CBL, which activated early endosomal antigen 1 (EEA1)-mediated endocytosis. TBR2 undergoes endocytosis through two pathways: EEA1-mediated endocytosis and lipid raft/caveolae-mediated pathways. The process of neddylation inhibits lipid raft/caveolae-mediated endocytosis, subsequently hindering TBR2 ubiquitination. These findings support the notion that leukemia patients have different types of C-CBL mutants, making it difficult to control cell arrest during the progression of leukemia^46^. C-CBL also plays a significant role in the neddylation of epidermal growth factor receptor (EGFR) tyrosine kinase, and it has been confirmed that C-CBL has multiple lysine residues that act as NEDD8 attachment sites. Although no further physiological responses were confirmed, the study highlighted the dual role of C-CBL as an E3 ligase and its participation in EGFR neddylation^47^. Another study investigating EGFR neddylation revealed that COP9 signalosome subunit 3 (Cops3), an E3 ligase, enhances stability through neddylation, in contrast with the effect of C-CBL. Desmoglein-1 (DSG1), found in the desmosome compartment, plays a crucial role in facilitating the deneddylation of the COP9 signalosome (CSN). This process effectively decreases the activity of EGFR by interacting with the Cops3 subunit of the CSN, thereby promoting epidermal differentiation^48^. Furthermore, MLN4924 treatment at nanomolar concentrations (30 nM–100 nM) induced EGFR dimerization, resulting in a significant increase in EGFR stability and activity. Consequently, the authors reported a marked increase in cancer proliferation and tumor sphere formation^49^.

The activation of the PI3K/AKT pathway by EGFR neddylation can have distinct effects independent of those associated with C-CBL^47^. These findings suggest that the neddylation of EGFR can elicit different responses on the same substrate through diverse mechanisms. As part of the EGFR family, the human epidermal growth factor receptor-2 (HER2) has also been found to be involved in neddylation in breast cancer. High levels of NEDD8 expression were detected in breast cancer patients. The authors verified that neddylation of HER2 increased its stability while inhibition of neddylation suppressed the growth of HER2-positive breast cancer cells^50^. Neddylation, which has an indirect yet notable influence on the receptor, has been reported to regulate Src homolog domain-containing phosphatase 2 (SHP2), a downstream component of signal regulatory protein α (SIRPα). In macrophages, SIRPα interacts with cluster of differentiation (CD47) on red blood cells, inhibiting phagocytosis. However, CD47 is also expressed in tumor cells, including colon cancer cells. The activation of SHP2 facilitated the binding of the CD47 ligand to the SIRPα receptor. Furthermore, neddylation of SHP2 reduces its activity, consequently inhibiting SIRPα/CD47 signaling and preventing macrophages from attaching to tumors^51^.

In cases where the classification is undefined but neddylation involvement is established, a follow-up study on EGFR neddylation revealed that vemurafenib, a BRAFV600E inhibitor used for colon cancers, has significantly improved efficacy when combined with pevonedistat (MLN4924) and cetuximab, an EGFR inhibitor^47,52^. Additionally, another study on dendritic cells revealed that pevonedistat treatment with lipopolysaccharide (LPS), a ligand for the Toll-like receptor (TLR), results in a reduction in LPS-induced proinflammatory cytokines^53^. Although the mechanisms underlying these findings are not yet fully understood, these studies serve as catalysts for further research on neddylation pathways. These results underscore the importance of ongoing research in this field.

## Neddylation in intracellular receptors

Intracellular receptors, a category of ligand-dependent transcription factors, include receptors for both steroid and nonsteroid hormones. Lipid-soluble hormones bind to these receptors, which are ligand-dependent transcription factors. The hormones traverse the plasma and nuclear membranes of target cells to bind with the receptors (see Fig. 2). Intracellular receptors are categorized into either Type I or Type II receptors according to their actions. Type I receptors, like steroid hormone receptors, initially reside in the cytoplasm and undergo translocation to the nucleus upon agonist stimulation, while Type II receptors are inherently located within the nucleus even in the absence of agonists^54^. To date, research on neddylation in Type I receptors has been far more actively pursued compared to Type II receptors. Owing to the absence of studies regarding neddylation in Type II receptors, we collectively highlight the latest reports related to Type II receptors.

### Steroid hormone receptors (Type I)

Type I intracellular receptors remain in an inactive state in the cytoplasm due to the binding of chaperone proteins. However, when a ligand binds with the receptor the chaperone proteins dissociate, facilitating the receptor to undergo a conformational change that enables the ligand to enter the nucleus. Notably, ligands such as progesterone, androgen, estrogen, and glucocorticoids play crucial roles in initiating various cellular processes in different physiological contexts^55^.

#### Canonical regulation by neddylation

Most of the existing reports on canonical regulation by neddylation predominantly revolve around the estrogen receptor, with many indicating indirect binding. However, a specific study identified ubiquitin-activating enzyme 3 (Uba3) as an interacting protein for both estrogen receptor alpha (ERα) and beta (ERβ). Uba3 is a catalytic subunit of the NEDD8-activating enzyme, and its interaction with estrogen receptors highlights its importance in the neddylation pathway. While direct binding of NEDD8 with either ERα or ERβ has not been confirmed, neddylation activity is crucial for Uba3-mediated suppression of ER transactivation, potentially restricting steroid hormone action depending on the disease context^56^. In a subsequent study by the same author, an in-depth investigation of Uba3 and ERα in breast cancer was undertaken. These findings demonstrated that impaired neddylation diminishes Uba3 activity, thereby impeding proteasomal degradation of ERα and consequently contributing to antiestrogen resistance in breast cancer^57^. In an entirely different context linked to breast cancer, estrogen-related receptor beta (ERRβ) has been identified as a substrate for the E3 ligase SCF complex, leading to cullin-dependent degradation. To validate the correlation of ERRβ overexpression with increased survival rates in breast cancer patients, the authors verified the increase in ERRβ expression following MLN4924 treatment, along with the upregulation of ERRβ target genes such as p21 and E-cadherin^58^. As a result, this led to decreased cancer cell proliferation and migration, strongly indicating that targeting ERRβ could be a promising therapeutic strategy for breast cancer treatment.

#### Noncanonical regulation by neddylation

Most investigations into noncanonical regulation by neddylation have focused predominantly on reports concerning the androgen receptor (AR). However, prior to discussing the AR, one study examined the ERα. A previous study revealed that neddylation impairment in breast cancer induced forkhead box class O 3a (FOXO3a) nuclear export, resulting in a reduction in ER expression. FOXO3a phosphorylation, catalyzed by serum and glucocorticoid-inducible kinase (SGK), triggers FOXO3a nuclear export. The authors discovered that MLN4924 treatment enhances SGK stability, leading to FOXO3a nuclear export and decreased ER transcription. As a result, this stabilization effectively suppressed the growth of ER-positive breast cancer in vitro and in vivo^59^.

In the context of AR, two studies on the role of neddylation yielded contradictory results. In one study, the authors confirmed the presence of NEDD8 binding sites in AR, specifically at lysine 475 and lysine 862. The breeding of tubercles (Bts), which are normally absent in female zebrafish, is induced in NEDD8 null female zebrafish, leading to disruptions in oogenesis and oocyte maturation. However, deletion of AR or treatment with the androgen antagonist flutamide alleviated this effect, suppressing Bts growth. Ultimately, the authors concluded that when NEDD8 fails to bind to AR the ligand dihydrotestosterone (DHT) binds to AR, thereby increasing its activity^60^. In contrast, another study focused on prostate cancer revealed a decrease in the mRNA levels of AR and AR-V7 (constitutively active variants), along with their targets kallikrein-related peptidase 3 (KLK3), FK506-binding protein 5 (FKBP5), and NKX3, following MLN4924 treatment and NAE knockdown. The authors further reported that combined treatment with MLN4924 and an AR antagonist significantly suppressed prostate cancer cell growth. These findings strongly suggest that neddylation plays a positive regulatory role in AR/AR-V7 transcription and highlight the potential of MLN4924 as a highly effective therapeutic agent^61^.

A recent report highlighted the involvement of neddylation in the breakdown of aggregated AR proteins. The majority of patients with spinal bulbar muscular atrophy (SBMA) are insensitive to AR, which is caused by the formation of polyglutamine (PolyQ) due to trinucleotide CAG repeat expansion in AR patients. The aggregation of the AR protein occurs as a result of the presence of PolyQ, along with the colocalization of heat shock proteins (Hsp70, Hsp90) and NEDD8. The binding of NEDD8 facilitates the recruitment of PA700 proteasome caps for protein degradation^62^. Ultimately, all studies on Type I steroid-type receptors have been categorized into canonical and noncanonical neddylation-regulated groups, with a primary focus on estrogen and androgen receptors. This underscores the importance of further investigations into other receptor types.

### Nuclear hormone receptors (Type II, nonsteroid receptors)

Type II intracellular receptors are located in the nucleus and can directly modify transcription without requiring translocation. Even in the absence of agonists they normally form heterodimers with other nuclear receptors. Examples include peroxisome proliferator-activated, retinoic acid, and thyroid receptors, which participate in diverse cellular processes across various physiological contexts^54^. A recent study revealed that noncanonical regulation by neddylation plays an essential role in adipogenesis by binding with PPAR-γ. The authors discovered that during adipogenesis NEDD8 is significantly induced in preadipocytes and binds with PPAR-γ, thereby promoting stabilization. Treatment with MLN4924 inhibits the E3 ligase activity of MDM2, leading to reduced stability of PPAR-γ and consequently decreased adipocyte differentiation. This intervention has proven to be a highly effective antiobesity therapeutic strategy in mice, preventing high-fat diet-induced obesity and glucose intolerance^63^. Unlike Type I receptors, Type II receptors have not been extensively studied in the context of neddylation, suggesting an untapped area ripe for further investigation.

## Concluding remarks

**Fig. 3:**
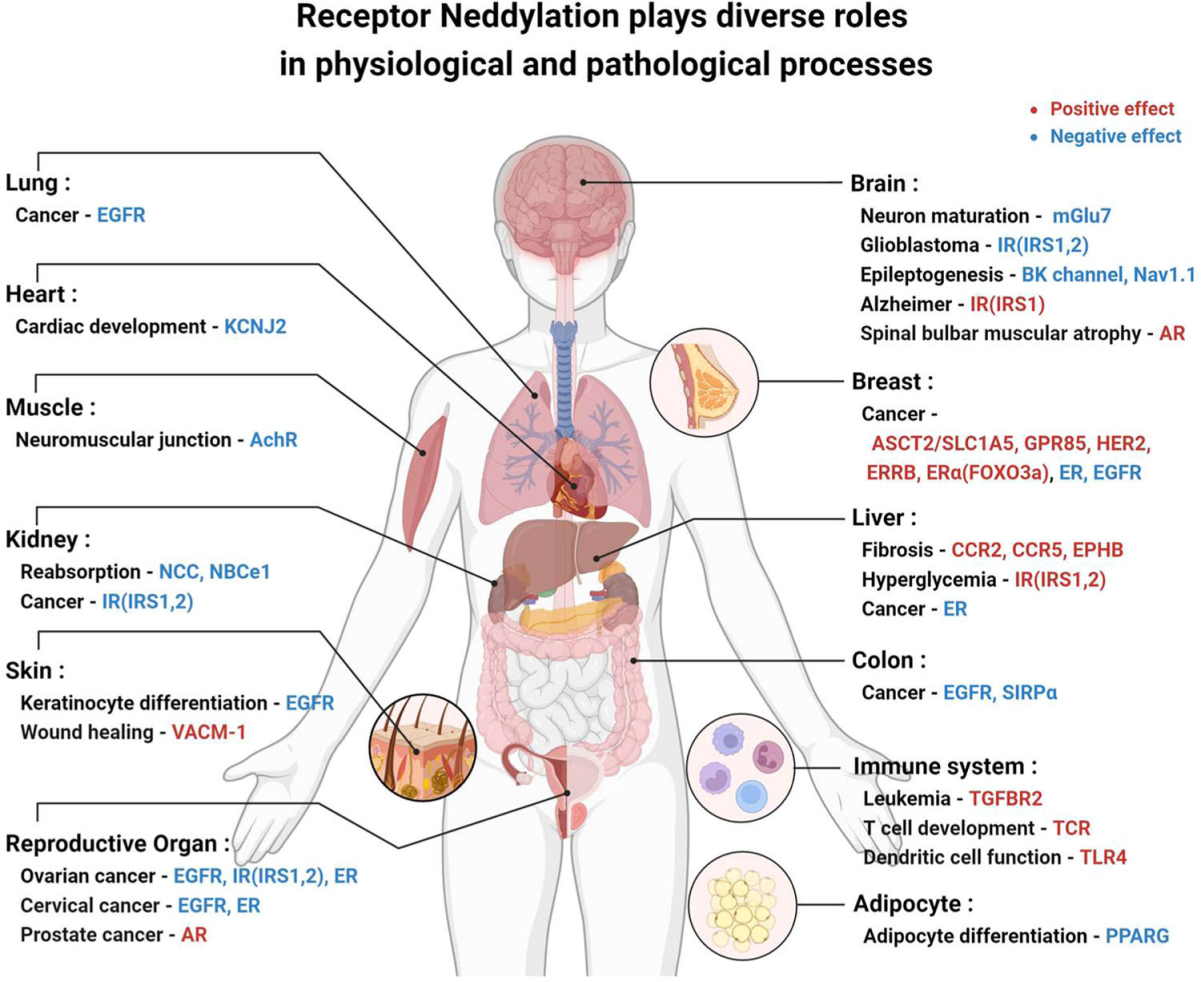
Receptor neddylation plays diverse roles in physiological and pathological processes. The response to receptor neddylation varies across different tissues and diseases. This diagram summarizes the effects of receptor neddylation investigated across various physiological responses, illustrating whether altering receptor function through neddylation inhibition has beneficial (positive) effects (highlighted in red) or harmful (negative) effects (highlighted in blue) on the respective tissues and diseases.

The regulation of cellular receptors by both canonical and noncanonical neddylation processes is crucial, as these receptors are key mediators of cellular communication and responses to environmental cues. Neddylation affects various proteins within both pathways, regulating receptor stability, signaling efficacy, protein aggregation, and transcriptional activity. This modification impacts a variety of receptors across different tissues leading to diverse disease manifestations. For example, neddylation of enzyme-linked receptors and Type I cytoplasmic receptors is well documented in cancer, while neddylation of other receptor types is associated with a range of conditions, including neurodegenerative diseases, obesity, and type 2 diabetes (Table 1, Fig. 3). Given the lack of studies addressing the role of neddylation in receptors and their related physiological responses, our aim is to provide valuable guidance by summarizing the functions of neddylation for each class of receptors and its physiological impacts across various tissues affected by diseases.

In recent years, significant progress has been made in the development of neddylation inhibitors, with some advancements in clinical trials. These inhibitors affect the stability and function of proteins regulated by neddylation, demonstrating their efficacy in targeting not only cancer but also other diseases associated with neddylation^64^. Considering that cellular receptors constitute the cornerstone of drug studies we believe that this review will contribute to advancing investigations in this area of development.
